# Supplementary material for: BRWD1 orchestrates epigenetic landscape of late B lymphopoiesis
Source: Nat Commun. 2018 Sep 24;9:3888. doi: 10.1038/s41467-018-06165-6 (PMC6155124; doi:10.1038/s41467-018-06165-6)
Supplement: Supplementary file 1 — Supplementary Information [file 41467_2018_6165_MOESM1_ESM.pdf]

Supplementary Information for

BRWD1 orchestrates epigenetic landscape of late B lymphopoiesis

Malay Mandal[1]*, Mark Maienschein-Cline[2], Patrick Maffucci[3], Margaret Veselits[1], Domenick E Kennedy[1], Kaitlin C McLean[1], Michael K Okoreeh[1], Sophiya Karki[3], Charlotte Cunningham-Rundles[4], Marcus R Clark[1]*

[1]Department of Medicine, Section of Rheumatology and Gwen Knapp Center for Lupus and Immunology Research, University of Chicago, Chicago, Illinois, USA.

[2]Core for Research Informatics, University of Illinois at Chicago, Chicago, Illinois, USA.

[3]Department of Research Biology, Genentech, South San Francisco, California, USA

[4]Immunology Institute, Icahn School of Medicine at Mount Sinai, New York, NY, USA; Division of Clinical Immunology, Department of Medicine, Icahn School of Medicine at Mount Sinai, New York, NY, USA.

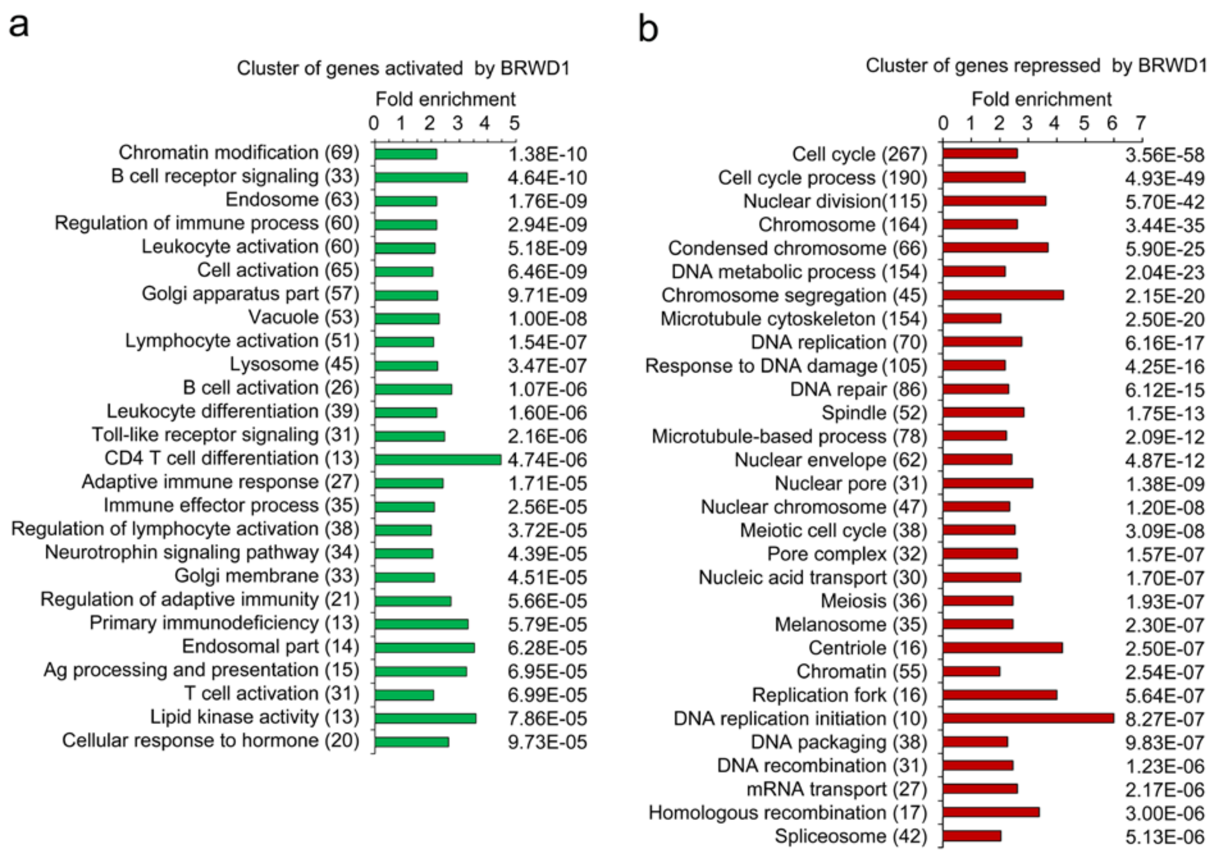

Supplementary Figure 1 BRWD1 enhances differentiation, and represses proliferation, transcription programs. **a, b** Ontology analysis (DAVID bioinformatics database) of gene clusters with lower (left panel, green) or higher (right panel, red) transcript abundance in *Brwd1*$^{-/-}$ small pre-B cells than in WT small pre-B cells. Only terms with FDR<5%, P<0.000075 and over $\log_2$ 2-fold enrichment were reported. The numbers in the parenthesis indicate the number of genes associated in that pathway.

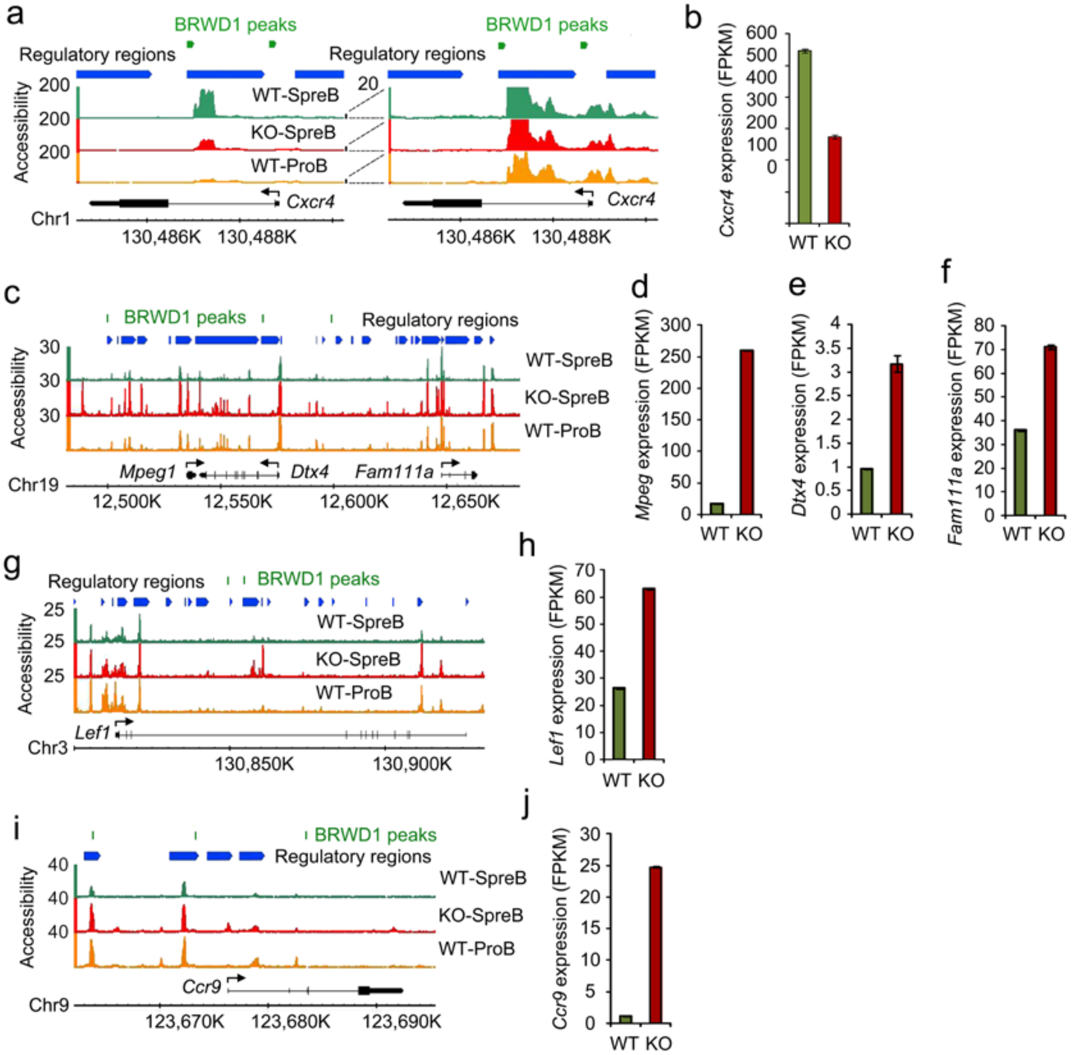

Supplementary Figure 2 BRWD1-mediated changes in enhancer accessibility and transcription. **a, b** Accessibility profile (**a**) and corresponding mRNA expression (**b**) of *Cxcr4* (upregulated during pre-B cell developmental progression) in WT pro-B, WT small pre-B and *Brwd1*$^{-/-}$ small pre-B cells. The enhancer of *Cxcr4* is ~1.6kb away from respective TSS. **c-j** Accessibility profiles (**c,g** and **i**) and corresponding mRNA expressions (**d,e,f,h** and **j**) of *Mpeg1, Dtx4, Fam111a* (**d-f**), *Lef1* (**g, h**) and *Ccr9* (**I, j**) (downregulated during pre-B cell developmental progression) in WT pro-B, WT small pre-B and *Brwd1*$^{-/-}$ small pre-B cells. The enhancers of *Mpeg1, Dtx4, Fam111a*, *Lef1* and *Ccr9* are respectively ~35kb (*Mpeg1*), ~7kb and 25kb (*Dtx4*), ~50kb, (*Fam111a*), ~35 and ~40kb (*Lef1*), ~4kb and 12.5kb (*Ccr9*) away from respective TSS.

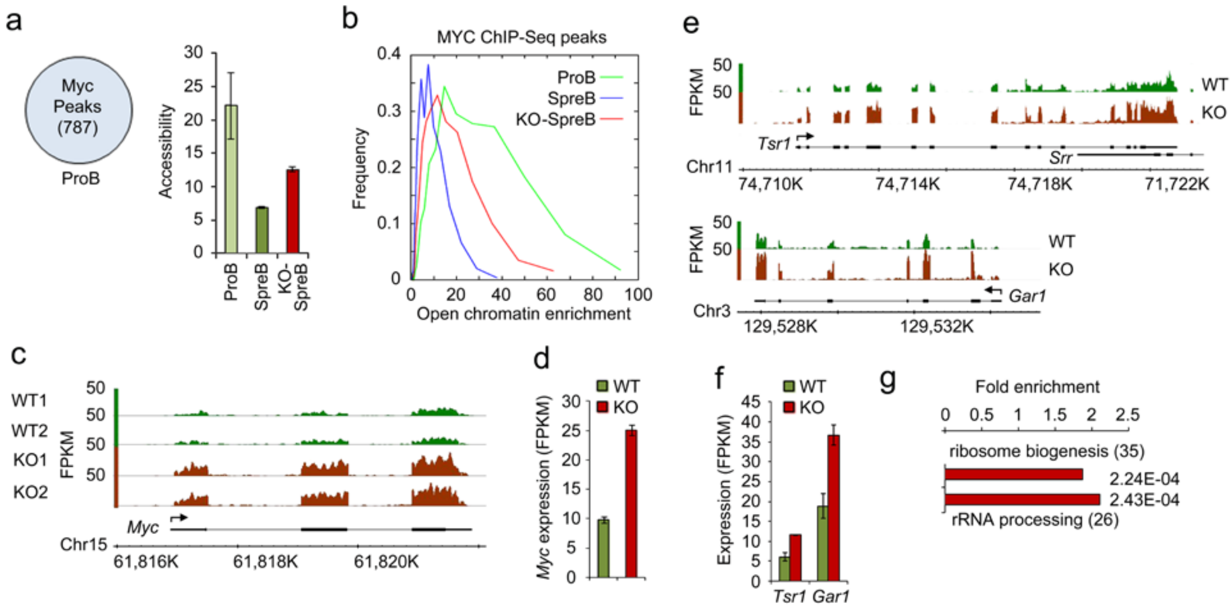

Supplementary Figure 3 BRWD1 represses Myc and its targets. **a** Number of MYC ChIP-Seq peaks in pro-B cells and the average accessibility of *Myc* binding sites in WT pro-B, WT small pre-B and *Brwd1*$^{-/-}$ small pre-B cells. **b** Histograms showing enrichment distribution of accessibility over WT pro-B MYC ChIP-Seq peaks in WT pro-B, WT small pre-B and *Brwd1*$^{-/-}$ small pre-B cells. **c** RNA-seq profiles of *Myc* gene (Fragments per kilobase of transcript per million mapped reads, FPKM) in WT and *Brwd1*$^{-/-}$ small pre-B cells. **d** Average expression (RNA-Seq) of *Myc* in WT and *Brwd1*$^{-/-}$ small pre-B cells. **e** RNA-seq profiles of MYC targets *Tsr1* and *Gar1* encoding products associated with ribosome biogenesis and rRNA processing. **f** RNA-Seq expression of *Tsr1* and *Gar1* in WT and *Brwd1*$^{-/-}$ small pre-B cells. **g** Gene ontology analysis (DAVID bioinformatics database) of MYC associated gene clusters enriched only in *Brwd1*$^{-/-}$ versus small pre-B cells.

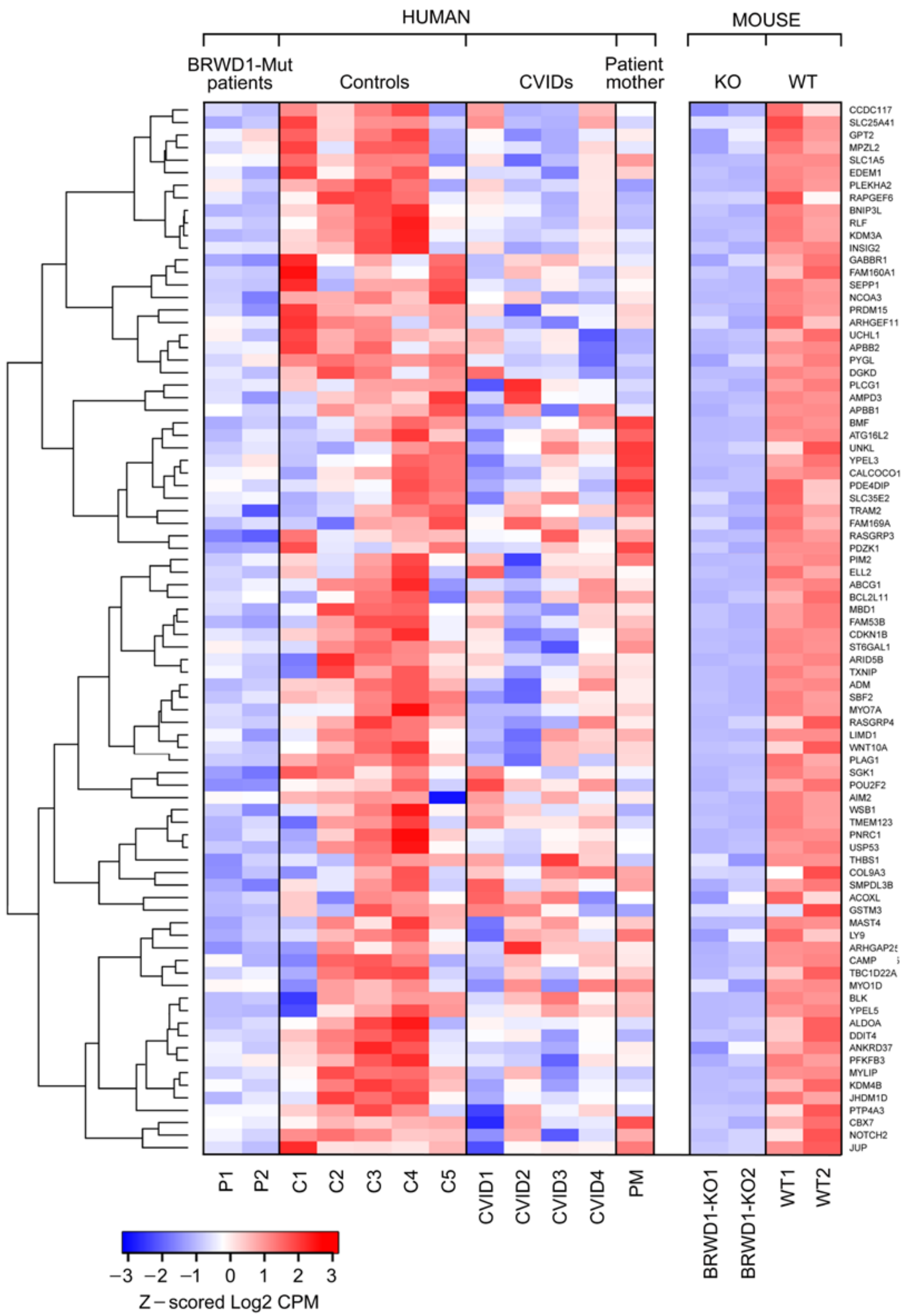

Supplementary Figure 4 BRWD1 upregulates similar genes in mice and humans. Heatmap of downregulated genes identified at $q<0.05$ in BRWD1-Mut patients and *Brwd1*$^{-/-}$ cells.

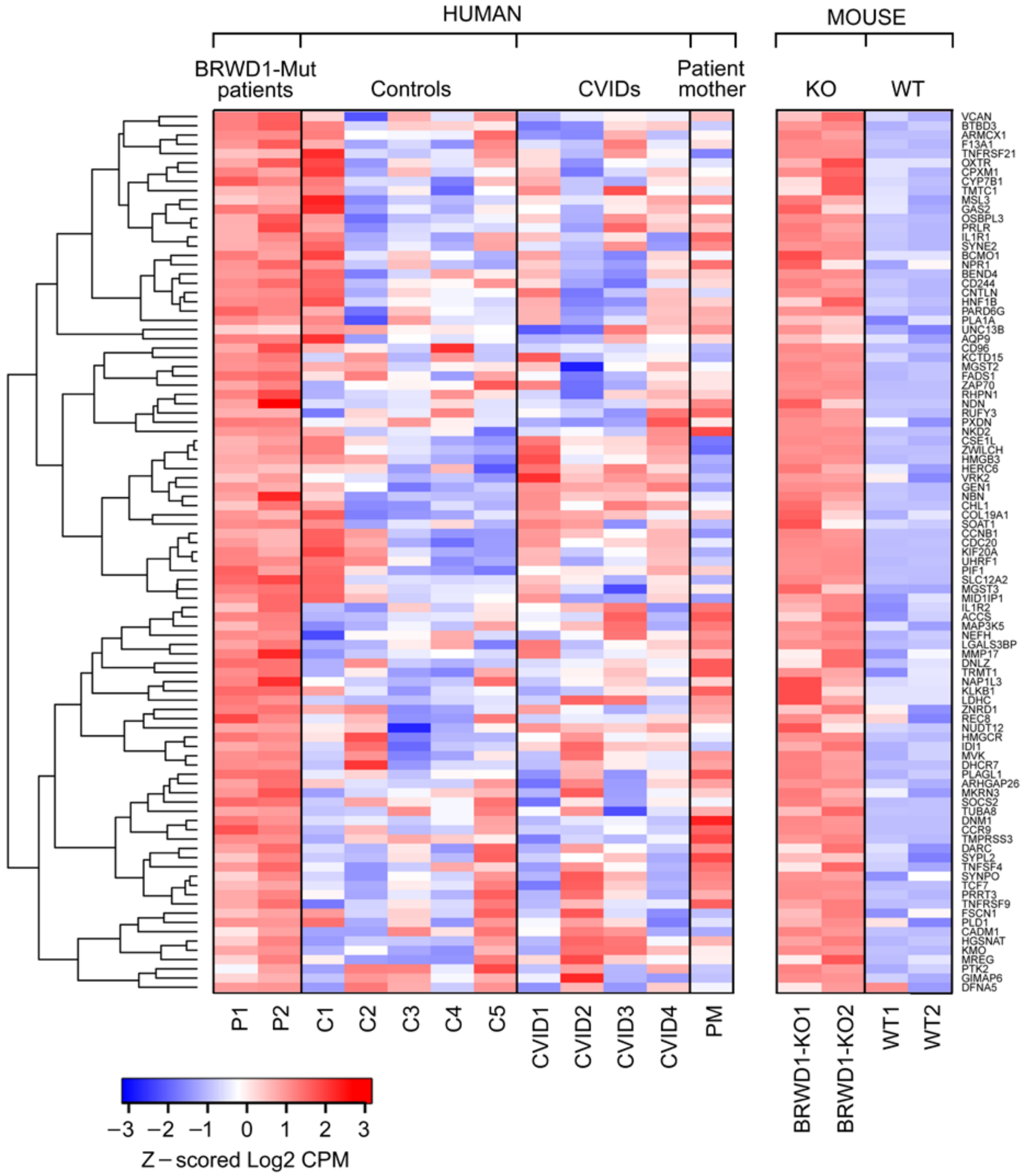

Supplementary Figure 5 BRWD1 represses the same set of genes in both humans and mice.

Heatmap of upregulated genes identified at q<0.05 in BRWD1-Mut patients and *Brwd1*$^{-/-}$ cells.

a

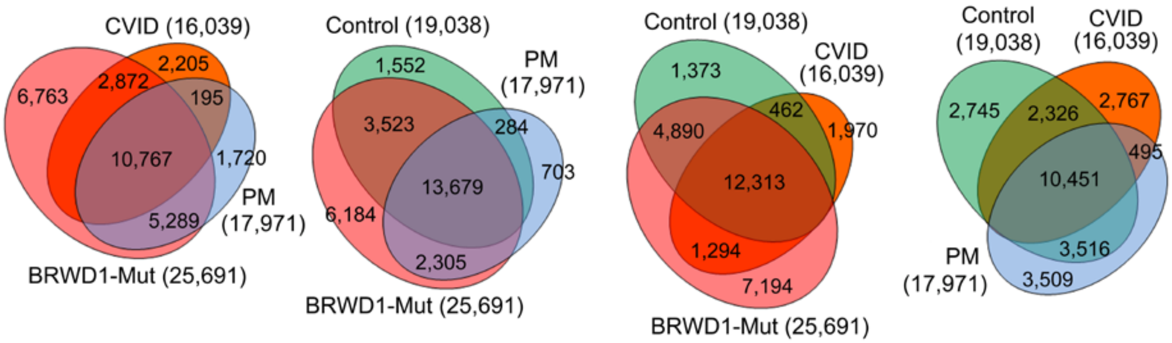

b

Known Transcription Factor Motifs	Motif Name	BRWD1-Mut to Controls		BRWD1-Mut to PM		BRWD1-Mut to CVIDs	
		Enrichment (+) /Depletion (-) (Log2 ratio)	P-value	Enrichment (+) /Depletion (-) (Log2 ratio)	P-value	Enrichment (+) /Depletion (-) (Log2 ratio)	P-value
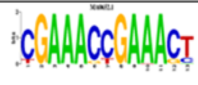	IRF	-1.10951	2.87E-116	-0.55115	1.98E-34	-0.24838	1.38E-37
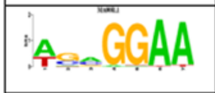	SPIB	-0.95847	1.66E-137	-0.56171	3.06E-12	-0.01873	5.48E-1
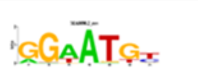	TEAD	1.404805	1.49E-93	0.29923	3.54E-14	0.504573	6.14E-35
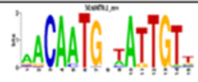	SOX1	1.298554	2.02E-55	0.56066	2.42E-12	0.361181	2.03E-13
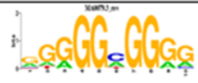	SP1/2	-0.64372	1.08E-43	-1.75131	1.151E-903	0.312582	1.76E-86
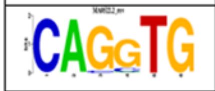	TCF3 (E12/E47)	-1.47763	1.503E-354	-0.68821	1.38E-23	-0.137263	1.26E-8
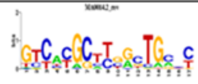	PAX5	-0.74275	6.17E-23	-1.64727	2.223E-347	-0.404939	1.72E-62

Supplementary Figure 6 Chromatin accessibility and enrichment of known transcription factor motifs. **a** 3-way overlap of open chromatin peaks (ATAC-Seq) in EBV transformed cells of BRWD1-mut hypogammaglobulinemia patient group (P1 and P2), control group (C1-5), unrelated common variable immunodeficiency group (CVID1-4) and patients' mother (PM). **b** Enrichment and Depletion of known transcription factor motifs in patient group (P1 and P2), compared to control group (C1-5), unrelated common variable immunodeficiency group (CVID1-4) and patients' mother (PM).

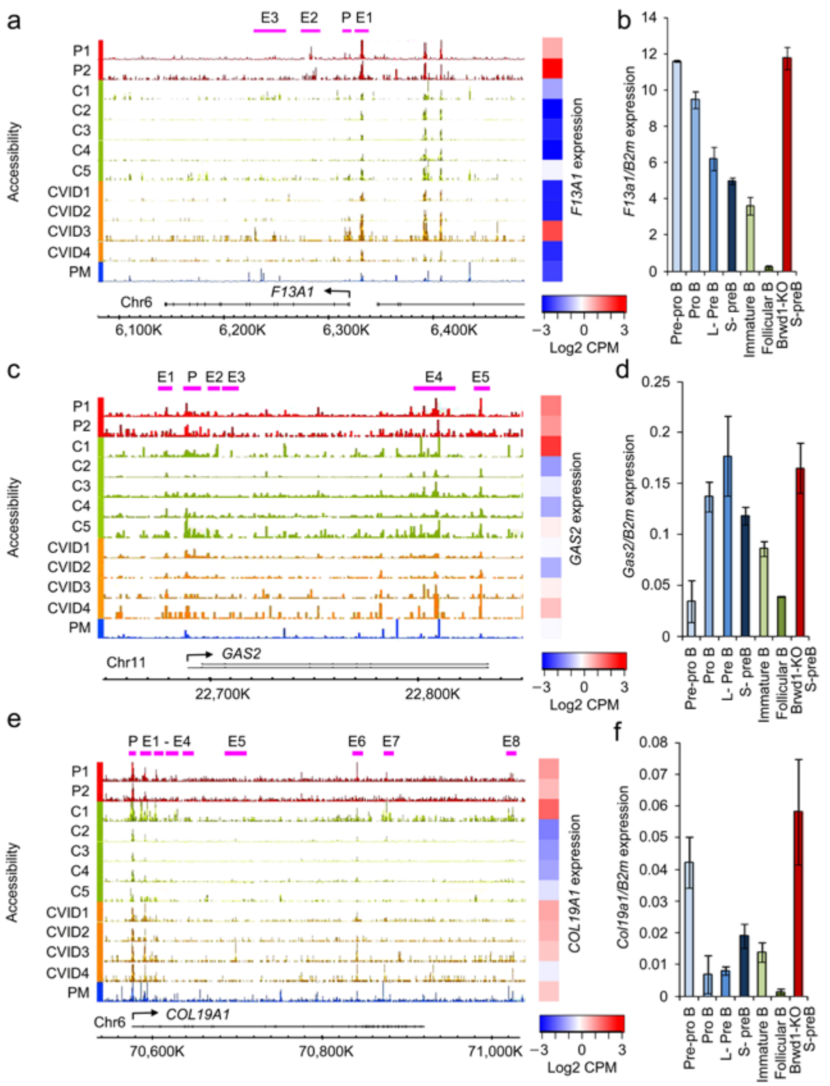

Supplementary Figure 7 Examples of BRWD1 mediated enhancer repression in humans and mice. **a** Accessibility status (left panel) of *BRWD1*-Mut patients (P1 and P2), controls (C1-5), unrelated CVID patients (CVID1-4) and PM with enhancer ($H3K4me1^{+}H3K27Ac^{+}$) profile in *F13A1* (Coagulation factor XIII A subunit) locus and heatmap of *F13A1* mRNA expression in the above samples (right panel). **b** Normalized expression of *F13a1* throughout B cell development stages from WT pre-pro-B to follicular B cells **c** Accessibility status of *GAS2* (Growth arrest specific 2) locus with enhancer profile (left panel) and heatmap of *GAS2* mRNA expression (right panel) in human samples described above. **d** Normalized expression of *Gas2* at different developmental stages of mouse B lymphopoiesis and in *Brwd1*$^{-/-}$ small pre-B cells. **e** Accessibility status of *COL19A1* (Collagen type XIX alpha 1) locus with enhancer profile (left panel) and heatmap of *COL19A1* mRNA expression (right panel) in human samples described above. **f** Normalized expression of *Col19a1* at different developmental stages of mouse B lymphopoiesis and in *Brwd1*$^{-/-}$ small pre-B cells.

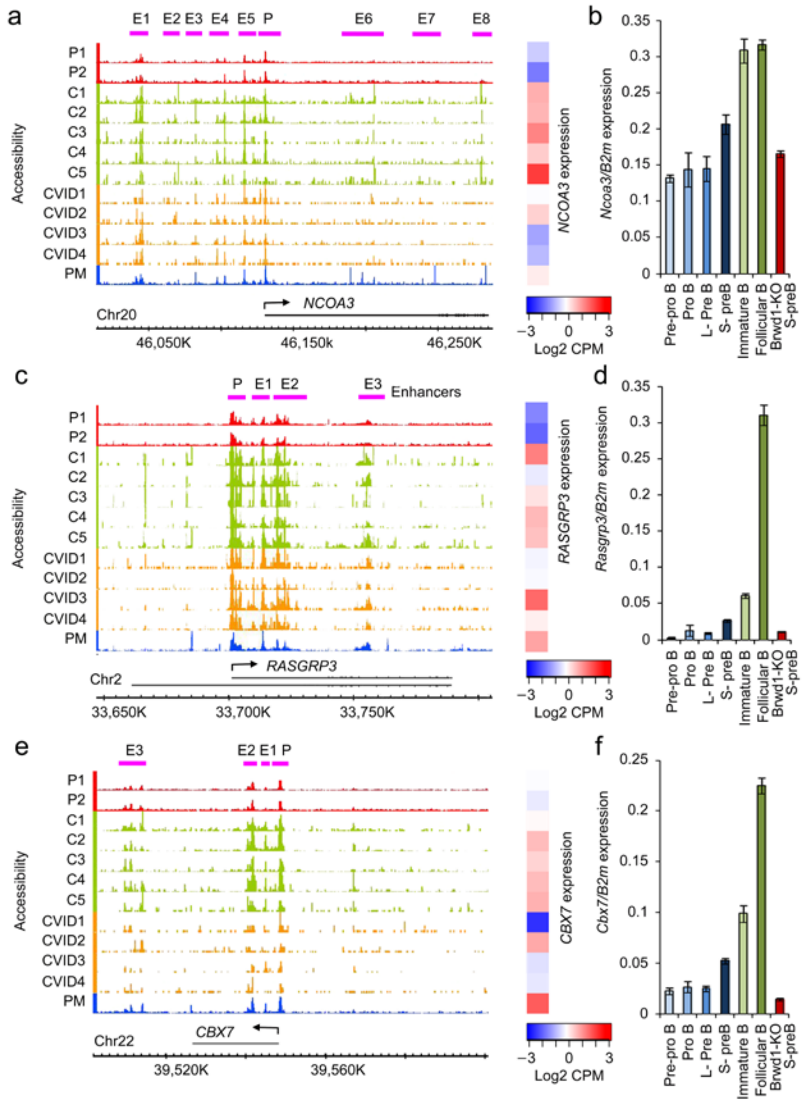

Supplementary Figure 8 Examples of BRWD1 mediated enhancer activation in humans and mice. **a** Accessibility status (left panel) of *BRWD1*-Mut patients (P1 and P2), controls (C1-5), unrelated CVID patients (CVID1-4) and PM with enhancer profile ($H3K4me1^+H3K27Ac^+$) in *NCOA3* (Nucelar receptor coactivator 3) locus and heatmap of *NCOA3* mRNA expression in the above samples (right panel). **b** Normalized expression of *Ncoa3* throughout B cell development stages from WT pre-pro-B to follicular B cells **c** Accessibility status of *RASGRP3* (Ras guanyl-releasing protein 3) locus with enhancer profile (left panel) and heatmap of *RASGRP3* mRNA expression (right panel) in human samples described above. **d** Normalized expression of *Rasgrp3* at different developmental stages of mouse B lymphopoiesis and in *Brwd1*$^{-/-}$ small pre-B cells. **e** Accessibility status of *CBX7* (Chromatin organization modifier box 7) locus with enhancer profile (left panel) and heatmap of *CBX7* mRNA expression (right panel) in human samples described above. **f** Normalized expression of *Cbx7* at different developmental stages of mouse B lymphopoiesis and in *Brwd1*$^{-/-}$ small pre-B cells.

Supplementary Table 1 Read and counts of RNA-Seq from purified WT and *Brwd1*$^{-/-}$ small pre-B cells.

Sample	Type	Raw reads - R1	Trimmed reads - R1	%	rRNA filtered reads - R1	%	Aligned reads	%
WT Small pre-B1	RNA-seq	30902952	29789458	96.4%	28394432	91.9%	27738257	89.8%
WT Small pre-B2	RNA-seq	27809486	26805184	96.4%	25614886	92.1%	25025409	90.0%
rna_ProB1	RNA-seq	30639854	29861436	97.5%	28993759	94.6%	28448198	92.8%
rna_ProB2	RNA-seq	27189999	26538050	97.6%	25661970	94.4%	25196166	92.7%
Brwd1-Mut Small pre-B1	RNA-seq	33763496	32563179	96.4%	31178810	92.3%	30565088	90.5%
Brwd1-Mut Small pre-B2	RNA-seq	35074875	33846468	96.5%	32492339	92.6%	31789288	90.6%

Supplementary Table 2 GO pathway analysis of upregulated and downregulated genes in *Brwd1*$^{-/-}$ small pre-B cells compared to WT small pre-B cells.

Gene down regualted in Brwd1-Mut compared to WT SpreB (genes activated by BRWD1)

Combine analysis; log2 fold change>2; p value < 0.000075 — same group not considered twice

Category	Term	Fold Enric	PValue	FDR	Count	Genes
GOTERM_	Chromatin modification (69)	2.218181	1.38E-10	2.53E-09	69	KDM6A, EZH1, BNIP3, INO80, CBX8, CBX7, KDM1B, WBP7, EPC1, EPC2, MLL5, PRMT6, TLK2, KDM5A, MLL3, KDM5B, KDM5C, KDM5D, SATB2, HDAC10, H
KEGG_PA1	B cell receptor signaling pathway (33)	3.273755	4.64E-10	5.71E-09	33	NFKBIE, NFKBIA, CD72, BTK, AKT1, FOS, RASGRP3, DAPP1, NFAT5, CD22, PIK3CA, PIK3AP1, PPP3CA, PIK3R3, CHUK, BLNK, PIK3R2, PIK3CG, BCL10, CR2
GOTERM_	Endosome (63)	2.209187	1.76E-09	2.54E-08	63	SLC9A7, CYTIP, RAB5B, CHMP5, NSG2, LGMN, CHMP7, VPS52, CD1D1, VPS33B, CD1D2, SLA, GRIPAP1, VPS11, ATP6V0D1, INSR, RAB21, ARHGAP27, VP
GOTERM_	Positive regulation of immune system proce	2.209754	2.94E-09	5.40E-08	60	TLR2, IL15, CD1D1, C1QC, CD1D2, KLHL6, HLX, ITCH, LBP, DAF2, H2-K1, BCL10, LYN, RELA, CD40, SOCS5, C1QA, C1QB, CD38, CD83, CD37, CD80, TNFSF1
GOTERM_	Leukocyte activation (60)	2.147868	5.18E-09	9.51E-08	60	XRCC4, ZBTB32, TLR1, IL15, CD1D1, CXCL12, CD1D2, MLL5, CXCR5, CXCR4, MS4A1, LBP, DLG1, RHOH, EGR1, BCL10, RELB, CD40, LIG4, WAS, PRKCD, DCL
GOTERM_	Cell activation (65)	2.06633	6.46E-09	1.19E-07	65	ZBTB32, XRCC4, TLR1, IL15, NFKB2, CD1D1, CXCL12, CD1D2, MLL5, CXCR5, CXCR4, MS4A1, LBP, LTB, DLG1, RHOH, EGR1, BCL10, RELB, CD40, LIG4, WAS,
GOTERM_	Golgi apparatus part (57)	2.218919	9.71E-09	1.41E-07	57	SLC9A7, AP4E1, SGMS2, TGOLN1, CHPF2, PIP5K1A, SGMS1, CBFA2T3, SLC35A1, TMF1, AP1S3, ST3GAL1, BLZF1, CHIC2, GOPC, GOLGA1, SPG21, GOLGA5
GOTERM_	Vacuole (53)	2.295822	1.00E-08	1.45E-07	53	SGSH, SLC36A1, LGMN, ATP6AP1, MYO7A, USE1, CD1D1, VPS33B, CD1D2, VPS11, MAN2B1, DPP7, IDUA, BCL10, ATG9A, 0610031J06RIK, LMBRD1, H2-D
GOTERM_	Lymphocyte activation (51)	2.105244	1.54E-07	2.84E-06	51	XRCC4, ZBTB32, IL15, CD1D1, CXCL12, CD1D2, CXCR5, CXCR4, MS4A1, RHOH, DLG1, EGR1, RELB, LIG4, CD40, PRKCD, WAS, DCLRE1C, CD86, GADD45G, P
GOTERM_	Lysosome (45)	2.23401	3.47E-07	5.02E-06	45	SGSH, SLC36A1, MFSD8, LGMN, MYO7A, USE1, ACP5, IFI30, CTSA, ACP2, PPT1, CD1D1, VPS33B, CD74, CD1D2, SLC11A2, SLC11A1, MAN2B1, VPS11, DPI
GOTERM_	B cell activation (26)	2.723475	1.07E-06	1.96E-05	26	XRCC4, IL7R, CXCR5, POU2F2, POLM, MS4A1, BCL3, BCL6, TCF3, BLNK, TRP53, CR2, IKZF1, SWAP70, MALT1, LIG4, CD40, PRKCD, FOXP1, HDAC5, DCLRE
GOTERM_	Leukocyte differentiation (39)	2.190562	1.60E-06	2.94E-05	39	XRCC4, SOX4, SFPI1, CACNB4, IL15, CD1D1, CBFA2T3, IL7R, CD74, CD1D2, DOCK2, POU2F2, POLM, BCL3, SPIB, BCL6, FLT3L, TRAF6, TCF3, CHUK, RHOH,
KEGG_PA1	Toll-like receptor signaling pathway (31)	2.485128	2.16E-06	2.66E-05	31	TLR1, TLR2, TIRAP, NFKBIA, TLR6, AKT1, IRAK4, FOS, PIK3CA, LBP, TRAF6, PIK3R3, MAP2K7, MAP2K6, CHUK, TRAF3, PIK3R2, PIK3CG, RELA, MAPK11, C
GOTERM_	CD4 T cell differentiation (13)	4.483123	4.74E-06	8.70E-05	13	IKZF1, IL4RA, TGFBR2, SOCS5, CD1D1, CD1D2, GM614, CD83, HLX, RIPK2, AP3D1, BCL6, IL2RG, RC3H1, SASH3
GOTERM_	Adaptive immune response (27)	2.438622	1.71E-05	0.000314	27	XRCC4, C3, NFKB2, TLR6, C1QC, CD74, SLC11A1, KLHL6, POU2F2, BCL3, BCL6, TRAF6, DLG1, DAF2, BCL10, CR2, SWAP70, RELB, LIG4, PRKCD, C1QA, C1Q
GOTERM_	Immune effector process (35)	2.107451	2.56E-05	0.00047	35	XRCC4, C3, FCNB, BNIP3, IL7R, C1QC, CD74, SLC11A1, MLL5, POU2F2, POLM, BCL3, LBP, TCF3, DAF2, DLG1, H2-Q2, BCL10, CR2, MSH3, SWAP70, ELANE,
GOTERM_	Regulation of lymphocyte activation (38)	2.002078	3.72E-05	0.000683	38	LST1, NFKBID, IL4RA, IL15, CD1D1, IL7R, TNFRSF4, CD74, CD1D2, STAT6, BLOC1S3, HLX, AP3D1, IL2RG, BCL6, ITCH, CSK, TCF3, RC3H1, DLG1, BCL10, IL2R
KEGG_PA1	Neurotrophin signaling pathway (34)	2.075668	4.39E-05	0.00054	34	NFKBIE, NFKBIA, FOXO3, AKT1, IRAK4, IRAK3, MAP3K1, CAMK2D, SH2B3, PIK3CA, CSK, TRAF6, PIK3R3, MAP2K7, CAMK2A, FRS2, PIK3R2, TRP53, PIK3C
GOTERM_	Golgi membrane (33)	2.12856	4.51E-05	0.000653	33	GLG1, AP4E1, SGMS2, CHPF2, SGMS1, CBFA2T3, ARFGEF2, SLC35A1, TMF1, ST3GAL1, AP1S3, MAN1A, GOLGA5, RAB21, GABARAPL2, IRGM1, ST6GAL1,
GOTERM_	Regulation of adaptive immune response (21	2.70958	5.66E-05	0.001039	21	H2-K1, H2-Q2, FCER2A, H2-M3, C3, IL4RA, TNFRSF13C, NDFIP1, CD40, SOCS5, CD1D1, IL7R, H2-Q7, CD1D2, H2-Q9, STAT6, SLC11A1, TNFSF13B, FCGR2B
KEGG_PA1	Primary immunodeficiency (13)	3.306823	5.79E-05	0.000712	13	RFX5, TNFRSF13C, CD40, IL7R, RFXANK, BTK, DCLRE1C, GM614, CD19, IKBKG, AICDA, IL2RG, CD79A, BLNK
GOTERM_	Endosomal part (14)	3.5347	6.28E-05	0.000909	14	RILP, SLC9A7, CHMP7, FIG4, CD63, CD1D1, APPL2, H2-DMB2, CD1D2, SLC11A2, SLC11A1, PIKFYVE, ARL8A, WDR44, RAB21
GOTERM_	Antigen processing and presentation of pept	3.251496	6.95E-05	0.001276	15	H2-K1, H2-Q2, H2-M3, H2-D1, UNC93B1, IFI30, H2-Q7, H2-DMB2, CD74, H2-Q9, SLC11A1, FCGR2B, H2-EB1, CTSE, H2-T23, TRAF6, 1500011B03RIK
GOTERM_	T cell activation (31)	2.092917	6.99E-05	0.001282	31	XRCC4, ZBTB32, SOX4, CACNB4, IL15, CD1D1, IL7R, CXCL12, ITGAM, CD74, CD1D2, SLC11A1, DOCK2, CXCR4, BCL3, DLG1, RHOH, TRP53, EGR1, H2-M3, I
GOTERM_	Lipid kinase activity (13)	3.596361	7.86E-05	0.001266	13	PIK3CG, PIK3C2A, PIK3C2B, PIP5K1C, PIP5K1A, PI4KB, PI4K2A, PIK3C3, PIKFYVE, PIK3CA, CERK, PIP4K2C, PIK3R2
GOTERM_	Cellular response to hormone stimulus (20)	2.616146	9.73E-05	0.001786	20	IRS2, SGK1, STAR, REN1, REN2, ADIPOR1, RHOQ, FOXO1, STXBP4, FOXO4, IRS1, LATS2, STAT3, AKT1, PHIP, EIF4EBP1, PIK3R3, INSR, GH, PIK3R2, NR1H

Gene upregualted in Brwd1-Mut compared to WT SpreB (genes repressed by BRWD1)

Combine analysis; log2 fold change>2; p value <0.000075 — same group not considered twice

Category	Term	Fold Enric	PValue	FDR	Count	Genes	
GOTERM_	Cell cycle (267)	2.622703	3.56E-58	6.63E-57	267	13588	KIFC1, RAD51C, S100A6, CROCC, AURKA, AURKB, PTTG1, CDCA8, SIN3A, CUL7, DDX11, RSPH1, INCENP, CDCA2, STAG3, H2AFX, NUP37, CDC
GOTERM_	Cell cycle process (190)	2.901618	4.93E-49	9.17E-48	190	13588	KIFC1, CEP72, RAD51C, SEPT2, DBF4, KNTC1, AURKB, PTTG1, CDCA8, SIN3A, RAD21, CDKN2A, OIP5, RSPH1, INCENP, CDCA2, STAG3, H2AFX
GOTERM_	Nuclear division(115)	3.632648	5.70E-42	1.06E-40	115	13588	KIFC1, SEPT2, KNTC1, AURKB, PTTG1, CDCA8, RAD21, OIP5, INCENP, CDCA2, NUP37, CDCA5, CCNA2, ASPM, STAG2, STAG1, CDCA3, ANAPC
GOTERM_	Chromosome (164)	2.609435	3.44E-35	5.07E-34	164	12504	ITGB3BP, XPO1, HMGN2, CBX4, KNTC1, CBX3, CBX2, RANGAP1, H1FX, AURKB, CBX1, HMGN5, CBX6, CBX5, PRIM1, CDCA8, HIST1H2BM, SIN
GOTERM_	Condensed chromosome (66)	3.709835	5.90E-25	8.70E-24	66	12504	MAD1L1, KNTC1, CBX3, MLH1, RANGAP1, NDE1, SEH1L, INCENP, STAG3, H2AFY, NUP37, TUBG1, TOP2A, ZW10, NUP133, DSN1, SGOL2, SGO
GOTERM_	DNA metabolic process (154)	2.195421	2.04E-23	3.80E-22	154	13588	RAD51C, XRCC2, PDGFB, DBF4, MORF4L2, 2310003H01RIK, PTTG1, MCM10, DNASE1L1, PRIM1, MUTYH, SIN3A, RAD21, DNTT, PRIM2, H2AFX
GOTERM_	Chromosome segregation (45)	4.219992	2.15E-20	3.99E-19	45	13588	RAD51C, NEK2, USP9X, A730008H23RIK, MLH1, CTCF, PTTG1, SPC25, RAD21, HJURP, SEH1L, INCENP, STAG3, NUP37, SKA1, CDCA5, TOP2A,
GOTERM_	Microtubule cytoskeleton (154)	2.058272	2.50E-20	3.69E-19	154	12504	FAM110A, KIFC1, GM6682, CEP72, SEPT2, PRC1, CEP76, CCDC85B, TUBB2A, CROCC, CEP78, DYNC2LI1, KNTC1, AURKA, AURKB, TTL, KIFC3, H
GOTERM_	DNA replication (70)	2.763972	6.16E-17	2.11E-15	70	13588	PDGFB, DBF4, MCM10, CDT1, PRIM1, CCNE2, CCNE1, MCM8, CDC45, MCM7, SIN3A, DNTT, PRIM2, POLL, CDC6, CCDC88A, RBBP4, POLH, DTL
GOTERM_	Response to DNA damage stimulus (105)	2.195768	4.25E-16	8.22E-15	105	13588	XRCC2, MORF4L2, 2310003H01RIK, PTTG1, MUTYH, RAD21, H2AFX, POLL, POLH, DTL, LIG1, NEIL3, USP1, POLE, TOPBP1, ESCO2, EEPD1, RAD
GOTERM_	DNA repair (86)	2.325009	6.12E-15	1.14E-13	86	13588	CLSPN, KIF22, NBN, XRCC2, MORF4L2, MLH1, 2310003H01RIK, PTTG1, FANCL, FANCM, MUTYH, RAD21, SLK, FANCI, NSMCE1, NSMCE2, H2A
GOTERM_	Spindle (52)	2.869269	1.75E-13	2.58E-12	52	12504	KIF22, KIFC1, SEPT2, PRC1, KNTC1, AURKA, AURKB, FAM83D, NDE1, INCENP, TUBG1, MYC, ASPM, ZW10, CDC6, KIF15, TPX2, NUSAP1, NUP
GOTERM_	Microtubule-based process (78)	2.218663	2.09E-12	3.88E-11	78	13588	KIF23, KIFC1, CEP72, GM6682, KIF22, KIF24, TUBB2A, CROCC, MLH1, TTL, KIFC3, HOOK1, KIF13A, KIF2C, NDE1, DYNLL2, SEH1L, KIFAP3, TUBE
GOTERM_	Nuclear envelope (62)	2.43722	4.87E-12	7.19E-11	62	12504	MAD1L1, XPO1, CBX3, EIF5A, NUP188, ANKLE1, HNRPLL, FANCL, SEH1L, NUP37, RANBP2, LBR, KPNB1, NUP133, NUP85, IPO9, SIGMAR1, NP
GOTERM_	Nuclear pore (31)	3.160124	1.38E-09	2.03E-08	31	12504	NXT1, XPO1, MAD1L1, NUP98, NUP93, EIF5A, NUP188, POM121, CSE1L, DDX19A, SEH1L, NUP50, NUP37, RANBP2, KPNB1, NUP133, RANBP1
GOTERM_	Nuclear chromosome (47)	2.336184	1.20E-08	1.77E-07	47	12504	ING2, NEK2, E2F6, TIPIN, POLA1, CBX3, MLH1, POLA2, CBX5, PRIM1, RPA1, SAP30, RPA2, PCGF2, RAD21, SMARCB1, INCENP, PRIM2, BUB1,
GOTERM_	Meiotic cell cycle (38)	2.534079	3.09E-08	5.74E-07	38	13588	RAD51C, NBN, NEK2, MRE11A, MLH1, PTTG1, RPA1, NUMA1, RSPH1, PSMC3IP, STAG3, MNS1, FBXO5, H2AFX, TUBG1, CLASP2, FANCA, LFN
GOTERM_	Pore complex (32)	2.636462	1.57E-07	2.32E-06	32	12504	NXT1, XPO1, MAD1L1, NUP98, NUP93, EIF5A, NUP188, POM121, CSE1L, DDX19A, SEH1L, NUP50, NUP37, RANBP2, KPNB1, NUP133, RANBP1
GOTERM_	Nucleic acid transport (30)	2.728076	1.70E-07	3.17E-06	30	13588	NXT1, XPO1, NCBP1, NUP93, EIF5A, NUP188, NXT2, POM121, DDX19A, SEH1L, RBM8A, NUP50, NPM1, NUP37, RANBP2, NUP133, RANBP17,
GOTERM_	Meiosis (36)	2.455268	1.93E-07	3.58E-06	36	13588	RAD51C, NBN, NEK2, MRE11A, MLH1, PTTG1, RPA1, RSPH1, PSMC3IP, STAG3, MNS1, FBXO5, H2AFX, CLASP2, FANCA, LFNG, STAG2, TRIP13
GOTERM_	Melanosome (35)	2.47653	2.30E-07	3.39E-06	35	12504	HSP90AB1, AHCY, PDIA3, ATP1B3, NAP1L1, PDIA6, PRDX1, ITGB1, CANX, CALU, ANXA6, SND1, FASN, RPN1, TMED10, HSPA8, RAB27A, P4HE
GOTERM_	Centriole (16)	4.183951	2.50E-07	3.69E-06	16	12504	CEP76, CROCC, CEP192, PCNT, CETN4, CETN3, CEP164, BIRC5, CEP55, NEDD1, PLK4, CNTROB, TUBD1, ODF2, TUBG1, CNTLN
GOTERM_	Chromatin (55)	2.00481	2.54E-07	3.75E-06	55	12504	KIF22, HMGN2, HP1BP3, CBX4, CBX3, CBX2, H1FX, CBX1, HMGN5, CBX6, CBX5, SAP30, HIST1H2BM, PCGF2, H2AFV, OIP5, INCENP, H2AFZ, H
GOTERM_	Replication fork (16)	4.00962	5.64E-07	8.32E-06	16	12504	NBN, BLM, E2F6, POLA1, CHEK1, POLA2, RFC5, PRIM1, RPA1, RPA2, RFC1, RFC2, PRIM2, PCNA, DNMT1, H2AFX
GOTERM_	DNA replication initiation (10)	6.001767	8.27E-07	1.54E-05	10	13588	CCNE2, CCNE1, CDC45, MCM7, POLA1, MCM2, MCM3, MCM4, MCM5, MCM6
GOTERM_	DNA packaging (38)	2.25809	9.83E-07	1.83E-05	38	13588	HIST1H4K, HIST1H2AF, HP1BP3, A730008H23RIK, HIST1H2AE, NAP1L1, H1FX, HIST1H2BM, NCAPH, SET, H2AFV, HJURP, CENPA, NCAPG2, H2
GOTERM_	DNA recombination (31)	2.48073	1.23E-06	2.28E-05	31	13588	RAD51C, NBN, XRCC2, BLM, UNG, MLH1, BANF1, RPA1, NSMCE1, PSMC3IP, NSMCE2, H2AFX, TRIP13, EXO1, RECQL4, MSH6, ICOSL, RAD51A
GOTERM_	mRNA transport (27)	2.613673	2.17E-06	4.04E-05	27	13588	NXT1, XPO1, NCBP1, NUP93, EIF5A, NUP188, NXT2, POM121, DDX19A, SEH1L, RBM8A, NUP50, NUP37, RANBP2, NUP133, RANBP17, MAGO
KEGG_PA1	Homologous recombination (17)	3.385956	3.00E-06	3.72E-05	17	5738	RAD51C, NBN, XRCC2, BLM, MRE11A, EME1, BRCA2, RAD54L, RAD50, RPA3, RAD51, POLD3, RPA1, RPA2, POLD1, POLD2, RAD54B
GOTERM_	Spliceosome (42)	2.037146	5.13E-06	7.57E-05	42	12504	RALY, NHP2L1, PPIL1, SNRPD3, SNRPD1, SNRPD2, SYNCRIP, SF3B5, SF3B4, PRPF19, RBM8A, HNRNPF, ISY1, U2AF1, LSM4, LSM3, PABPC1, LS

Supplementary Table 3 Read and counts of WT pre-proB, pro-B, large pre-B, small pre-B and Immature B cell ATAC-Seq.

Sample	Raw reads - R1	Raw reads - R2	Trimmed reads - R1	%	Trimmed reads - R2	%	Complete pairs after trimming	%	Aligned reads	%
Pre-ProB1	106501243	106501243	106077361	99.6%	105298138	98.9%	104062148	97.7%	102321987	96.1%
Pre-ProB2	114941632	114941632	114458755	99.6%	112924388	98.2%	106575886	92.7%	106890760	93.0%
ProB1	94922732	94922732	94499455	99.6%	93313214	98.3%	92071301	97.0%	91462304	96.4%
ProB2	105243429	105243429	104837721	99.6%	103600641	98.4%	102334192	97.2%	102096421	97.0%
Large preB1	133856866	133856866	133234394	99.5%	130527526	97.5%	123556090	92.3%	127888743	95.5%
Large preB2	126302810	126302810	125785509	99.6%	123413840	97.7%	116356666	92.1%	120312860	95.3%
Brwd1-Mut small pre-B1	86821861	86821861	86710495	99.9%	85797411	98.8%	83726404	96.4%	81520428	93.9%
Brwd1-Mut small pre-B2	96234426	96234426	96048959	99.8%	95175630	98.9%	92736672	96.4%	90880397	94.4%
Small pre-B1	70156781	70156781	70066574	99.9%	69455097	99.0%	67865040	96.7%	66158599	94.3%
Small pre-B2	83648534	83648534	83511078	99.8%	82600240	98.7%	80604130	96.4%	78692325	94.1%
ImmatureB1	169133991	169133991	167759884	99.2%	160067883	94.6%	153504702	90.8%	160678285	95.0%
ImmatureB2	158197780	158197780	156862546	99.2%	150894002	95.4%	146548158	92.6%	150119064	94.9%

Supplementary Table 4 Comparison of BRWD1 ChIP-Seq peaks with ATAC-seq of WT and *Brwd1*$^{-/-}$ small pre-B cells.

P-IDR (1843 BRWD1 peaks)						
Range	# peaks - UP	# peaks - DOWN	# peaks - No change	UP ATAC	DOWN ATAC	No change ATAC
**0bp-0bp**	34	187	70	34	191	70
**0bp-5kb**	283	387	406	360	538	523
**0bp-100kb**	1227	1165	1455	4564	4461	7013
**0bp-200kb**	1456	1415	1601	7999	7628	12211
**0bp-500kb**	1700	1678	1777	15252	14568	23502
**0bp-5Mb**	1842	1843	1842	27240	26090	41343

Total:

UP ATAC 27256

DOWN ATAC 26143

No change ATAC 41380

Change based on FDR < 0.05 and direction of change: UP = higher in *Brwd1*$^{-/-}$

P-hi (723 BRWD1 peaks; P<.0000001)						
Range	# peaks - UP	# peaks - DOWN	# peaks - No change	UP ATAC	DOWN ATAC	No change ATAC
**0bp-0bp**	10	116	21	10	104	21
**0bp-5kb**	91	195	146	117	228	185
**0bp-100kb**	440	476	553	1672	1841	2805
**0bp-200kb**	531	562	601	3156	3286	5176
**0bp-500kb**	664	664	693	7004	6797	11192
**0bp-5Mb**	720	722	720	25079	24362	38615

Supplementary Table 5 Clinical characteristics of *BRWD1*-mutant hypogammaglobulinemia patients.

	Year Born	Age diagnosis made	Age symp-toms	SEX	ASSOCIATED CONDITIONS notes	Unusual Infections	Autoimmunity	Hepatitis	Lung Disease	Gastrointestinal	Granuloma	Splenectomy
Patient 1	98.0	14		M	Giardia	Giardia						
Patient 2	92.0	16	14	M	Weakness and diarrhea, wt loss/ TPN for a while/ lo vit A/D/E/fe	Giardia/ norvirus				GI		
Patient 3	73	40	17	M	sinus infections/pna x 2 ; diarrhea							
Patient 4	86.0	8	12	M	campylobacter/ leg ulcers	camphlybacter						

	IGG (nl 600-1600 mg/dl)	IGA (nl 79- 230 mg/dl)	IGM (50-300 mg/dl)	T% only (57-75%)	B% (6-29%)	SWMB* (13- 28)	CD4/CD8	ABS CD4 (490-1700)
Patient 1	159	<5	19	83.0	12.0	0.4		
Patient 2	110	69	11	70.0	19.0	0.1	1.7	320.0
Patient 3	77	<1	<1	57	10	1.6		909
Patient 4	50	<5	<5	98.0	0.0	0.0	0.6	288.0

*isotype switched memory B cells-as a percent of total B cells

Supplementary Table 6 List of probes and primers

**Quantitative PCR**	
B-2 Microglobulin-F	5’-AGACTGATACATACGCCTGCA-3’
B-2 Microglobulin-R	5’-GCAGGTTCAAATGAATCTTCA-3’
Ikzf3-Fw	5’-GACACGTGCCCTATGACAACAGCAG-3’
Ikzf3-Rev	5’-GCATGCGTAGTTGCAGAGGTGACA C-3’
Myc-Fw	5′-GCCCCCAAGGTAGTGATCCT-3′
Myc-Rev	5′-GTGCTCGTCTGCTTGAATGG-3′
Irf4-Fw	5’-CTACCCCATGACAGCACCTT-3’
Irf4-Rev	5’-CCAAACGTCACAGGACATTG-3’
Yy1-Fw	5’-CAGAAGCAGGTGCAGATCAGACCCT-3’
Yy1-Rev	5’-GCACCACCACCCACGGAATCG-3’
Pax5-Fw	5'-CGCGTGTTTGAGAGACAGCACTACT-3'
Pax5-Rev	5'-GTCTCGGCCTGTGACAATAGGGTAG-3'
Tcfe2a-Fw	5'-TCCTTTGACCCTAGCCGGACATAC-3'
Tcfe2a-Rev	5'-CCAACACTGGTGTCTCTCCCAAAG-3'
**Sanger Sequencing for *BRWD1* gene variations patients and their healthy relatives**	
c.1838A>G-Fw	5’-ACGTCCTAATTGTCATAAGTG-3’
c.1838A>G-Rev	5’-CAAGTAATAGTTATCAGCTGTG-3’
c.2456dupA-Fw	5’-CCACTGGATTGCACAAGTATACAG-3’
c.2456dupA-Rev	5’-GGAAACTTGACGGAATGCCC-3’
c.2567+4T>C-Fw	5’- CTGTTGAGATGAAGGTGACGTG-3’
c.2567+4T>C-Rev	5’- GGGATGTAGTGAAGAAGTTTGC-3’
**Short Hairpin RNA Constructs for Human *BRWD1* (hBRWD1)**	
shRNA-hBRWD1-40 (Target Sequence)	5’-ACCCAGTATTTCAAGAGTCAC-3’
shRNA-hBRWD1-40 (97mer)	5’-TGCTGTTGACAGTGAGCGAACCCAGTATTTCAAGAGTCACTAGTGAAGCCACAGATGTAGTGACTCTTGAAATACTGGGTGTGCCTACTGCCTCGGA-3’
shRNA-hBRWD1-197 (Target Sequence)	5’-CCAAATCTTGTGGAGATACAT-3’
shRNA-hBRWD1-197 (97mer)	5’-TGCTGTTGACAGTGAGCGCCCAAATCTTGTGGAGATACATTAGTGAAGCCACAGATGTAATGTATCTCCACAAGATTTGGTTGCCTACTGCCTCGGA-3’
