# Supplementary material for: BRWD1 orchestrates epigenetic landscape of late B lymphopoiesis
Source: Nat Commun. 2018 Sep 24;9:3888. doi: 10.1038/s41467-018-06165-6 (PMC6155124; doi:10.1038/s41467-018-06165-6)
Supplement: Supplementary file 3 — Description of Additional Supplementary files [file 41467_2018_6165_MOESM3_ESM.pdf]

Supplementary data:

Downregulated in Brwd1-Mut compared to WT (3405 genes)
